# Enhanced Wound Healing after Leiomyoma Enucleation

**Published:** 2018-01

**Authors:** Masood Amini, Mohammad Hassan Hashemizadeh, Seyedeh Leila Poorbaghi

**Affiliations:** 1 Laparoscopy Research Center, Shiraz University of Medical Sciences, Shiraz, Iran;; 2 Health Policy Research Center, Shiraz University of Medical Sciences, Shiraz, Iran;; 3 Obesity Prevention and Treatment Research Center, Shiraz University of Medical Sciences, Shiraz, Iran

**Keywords:** Video assisted thoracoscopic surgery, Leiomyoma, Esophageal sleeve calibration tube

## Abstract

Leiomyoma is a rare esophageal tumor that constitutes less than 1% of esophageal malignancies. It should be removed when diagnosed even if asymptomatic. This study presents two cases of esophageal leiomyoma in 38 and 62 years old men who presented with dysphagia and evaluated for diagnosis and location of related tumors. Patients were clinically examined and upper gastrointestinal endoscopy, chest computerized tomography scan, barium swallow and endoscopic ultrasonography were performed. The masses were diagnosed and both patients underwent 3-port right videothoracoscopic enucleation of esophageal leiomyoma with sleeve calibration tube (SCT) assistance. SCT was used to protrude out the mass from surrounding tissues. Also immunohistochemistry was done after mass enucleation. Two patients were operated routinely without any unpredictable events by help of SCT assistance. Mass size was 3.5×2.5×1 cm in one case and 1.5×1×0.5 cm in another patient. Positive smooth muscle antigen, desmin and ki67 and negative CD34, CD117 and S100 in both cases were obtained in immunohistochemistry. Patients were followed after surgery for 3 months and no complications were detected in none of them. Thoracoscopic enucleation of esophageal leiomyoma is a safe and feasible procedure which can decrease hospitalization and operation time. Based on our findings, the use of esophageal SCT help to detect smaller tumor without need of intra-operative endoscopy, facilitates separation of the tumor mass from both esophageal mucosal and muscular layers, and may prevent perforation. Finally, use of SCT makes the operation faster and safer.

## INTRODUCTION

Morgagni described leiomyoma in 1761 and then Munro was reported esophageal leiomyoma in 1797.^1^^,^^2^ Also, the histological characteristics of leiomyoma was described in 1863 by Virchow.^2^ Leiomyoma constitutes more than 50% of benign esophageal tumors. It is, however, a rare condition with an overall incidence of 8-43 per 10000 in autopsy series.^3^^,^^4^ The leiomyoma was found in an intramural position within the esophageal wall (97%), true polypoid lesions (1%) and extending extra-esophageal as mediastinal out growths (2%). It is estimated that 10% of intramural leiomyoma was reported to have grown in a circumferential way, almost surrounding the esophageal lumen.^3^^,^^5^

About half of the patients with leiomyoma are asymptomatic; this may be more so in cases of lesions found incidentally at autopsy. The location was in lower third of the esophagus (56%), in the middle third (33%), in the upper third (11%) and small percentage at the gastro-esophageal junction (GEJ).^5^ They vary in size and shape from few mm to near 30 cm in diameter.^6^ Approximately half of the patients are asymptomatic, but when symptoms occur they comprise dysphagia, retrosternal pain, heartburn and weight loss.^3^^,^^4^ Diagnosis is usually made on esophagogram, chest X-ray, computerized tomography (CT) scanning and magnetic resonance imaging (MRI). Recently transesophageal echo probe has been widely used for pre-operative diagnosis.^3^^,^^4^^,^^7^

Leiomyoma should be removed unless there are specific contraindication. The majority can be removed by simple enucleation. The mortality rate associated with enucleation is low and success in relieving the dysphagia is near 100%. Large lesions or those involving the GEJ may require esophageal resection.^1,3^ Here, we describe two cases of symptomatic leiomyoma of the mid esophagus, resected after right videothoracoscopic enucleation with sleeve calibration tube (SCT) assistance.

## CASE REPORT

**Fig. 1:**
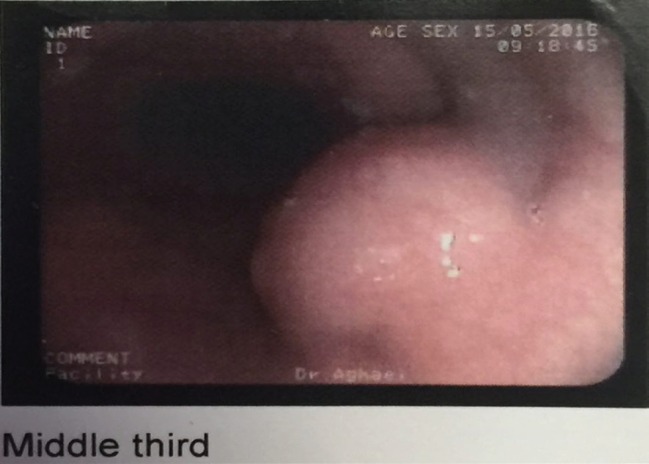
Upper gastrointestinal endoscopy in case 2. Smooth, submucosal mass in esophagus at 15 cm of incisures was detected.

**Fig. 2:**
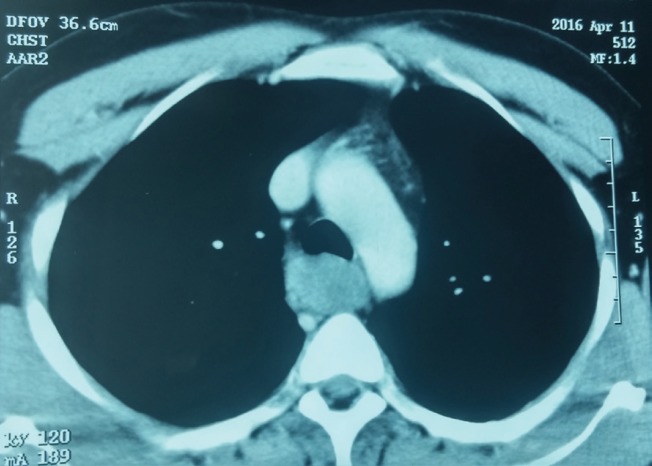
Well-defined solid homogeneous mediastinal mass in chest CT scan of case 1.

**Fig. 3:**
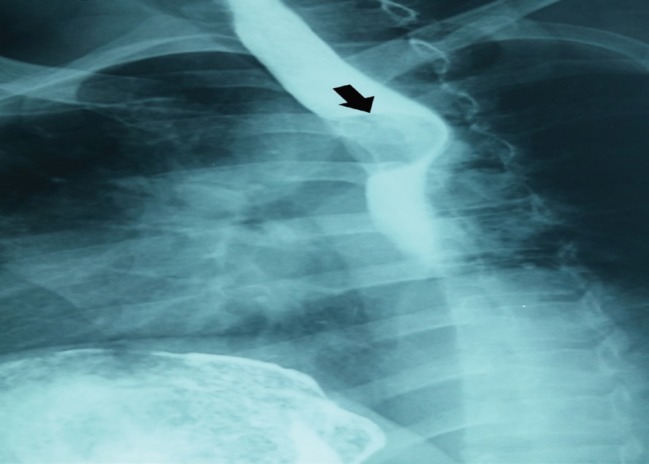
Smooth, semilunar filling defect in upper esophagus of case 1 after barium swallow which shown by arrow.

**Fig. 4:**
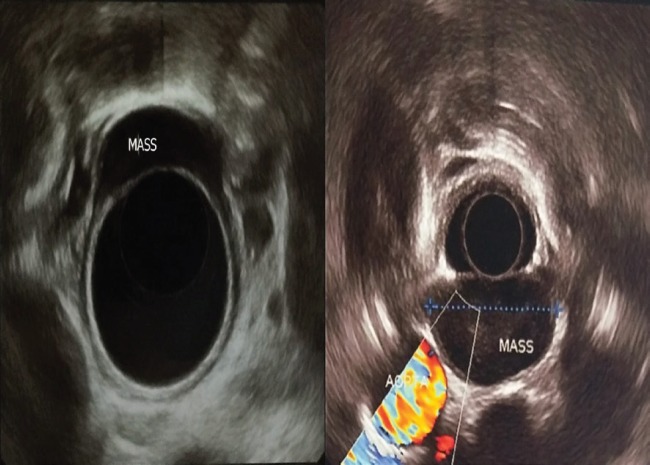
Endoscopic ultrasonography of case 1 (left) and case 2 (right). Left, 3.2 cm, hypoechoic, well capsulated, slightly heterogeneous mass from muscularis propria; Right, 1 cm, hypoechoic, and well capsulated, heterogeneous mass from muscularis propria (Figure 4, right).

The characteristic features of two patients are shown in Table 1. Diagnostic upper GI endoscopy (Figure 1), chest CT-scan (Figure 2), barium swallow (Figure 3) and endoscopic ultrasonograpy (Figure 4) were performed for both patients. After induction of general anesthesia and double-lumen endotracheal intubation, the operations were made in the left lateral decubitus position and the patients were rotated 20° to the front. A 10-mm port placed in the sixth intercostal space along the posterior axillary line. Introduce the 10-mm 30° scope. A second 10-mm port placed in the third intercostal space in the mid-axillary line. The third 5-mm port in the inter-scapulovertebral line, just over the tumor was inserted. Then left lung ventilation was done. The large mass of the case 1, easily found but in the case 2 which has small one, it was not seen. After passing of the SCT 40 F, mass was protrude out from the esophagus below the azygos vein. Then with a longitudinal mediastinal pleural incision, esophagus muscle was seen.

**Table 1:** Variables of 2 cases before and after operation.

**Variables**	**Case 1**	**Case 2**
Pre-operation		
Age (year)	38	62
Symptoms	Progressive dysphagia and retrosternal pain 3 months	Dysphagia for one month
Clinical examination	Normal	Normal
Biochemical parameters	Normal limits	Normal limits
Hematological parameters	Normal limits	Normal limits
Upper GI endoscopy	One large submucosal mass in esophagus at 25 cm from the incisuras.	Smooth, submucosal mass in esophagus at 15 cm of incisuras (Figure 1)
Chest CT scan	Well-defined solid homogeneous mediastinal mass parallel to the middle of the esophagus (Figure 2)	NP
Barium swallow	Smooth, semilunar filling defect that moves with swallowing (Figure 3)	NP
Endoscopic US	3.2 cm, hypoechoic, well capsulated, slightly heterogeneous mass from muscularis propria at 15 cm from the incisuras (Figure 4, left)	1 cm, hypoechoic, and well capsulated, heterogeneous mass from muscularis propria, at 15 cm from the incisuras (Figure 4, right).
Mass location	Upper mid-thoracic esophagus at supra-azygos vein	Mid-thoracic esophagus at infra-azygos vein
Post-operation		
Mass size (histopathology)	3.5×2.5×1 cm	1.5×1×0.5 cm
Immunohistochemistry	Positive for SMA, desmin and ki67 Negative for CD34, CD117 and S100	Positive for SMA, desmin and ki67 Negative for CD34, CD117 and S100

NP: not performed; SMA: smooth muscle antigen

The esophageal muscular layer was opened. The tumor was dissected away from the esophageal mucosa by blunt dissection without use of cautery (in order to avoid the risk of perforation due to a delayed burn injury). At this point, the tumor was completely enucleated by SCT assistance and anchoring of mass by silk stitch in case 1. In this case, we were not successful to pass the SCT in early operation before mass excision but was passed it after mass enucleation. The esophageal mucosa of case 1 was perforated during tumor enucleation and repaired by polydioxanone (PDS) 4-0. Next, the esophageal wall and mediastinal pleura were repaired with separate stitches of PDS 4-0 suture. Leakage test confirmed by insufflating methylene blue into the esophageal lumen through the SCT while distal site of operation was obstructed. 

**Fig. 5:**
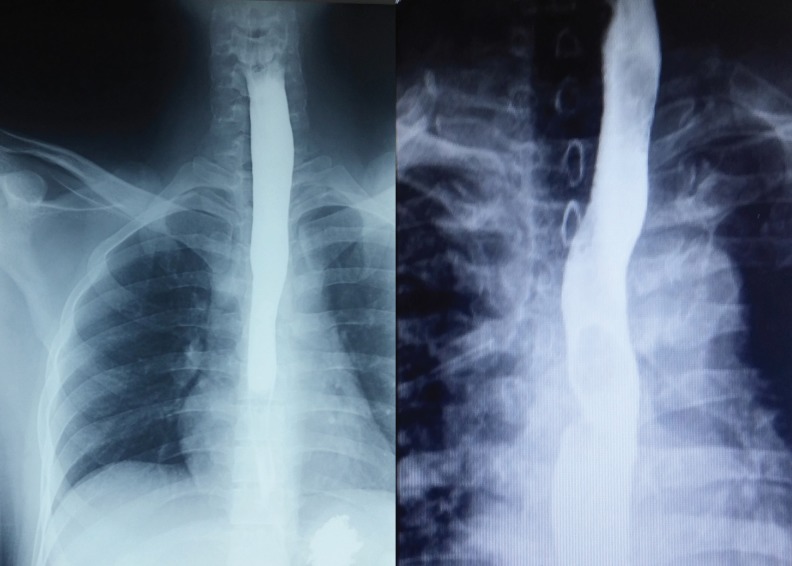
Normal barium swallow which done on 3^rd^ and 1^st^ days post-operation for case 1 (left) and case 2 (right), respectively.

The chest tube (No. 28) was placed along the mediastinal pleura over the reconstruction site. The lung was inflated to ensure complete expansion and the drain was secured to the skin with a silk stitch. Closure was performed with interrupt nylon 3-0 sutures and dressing was applied. Patients had an uneventful post-operative recovery. The naso-gastric tubes (NGT) were removed post-operation. In case 1 due to esophageal perforation fluids were started on day 4 post-operation. Case 2 was operated, identical to procedure of case 1, but mass was detected in middle thoracic esophagus, after passing of SCT 40 F and no esophageal perforation occurred. The patient had good post-operative recovery without problems. Barium swallow was done on 1^st^ and 3^rd^ days post-operation in case 2 and 1, respectively, which were normal (Figure 5). Both patients were asymptomatic, tolerating feeds and are on regular monthly follow up for 3 months.

## DISCUSSION

Leiomyoma is the most common benign neoplasm of the oesophagus accounting for 1% of all oesophageal tumors.^6^ They are usually solitary, but multiple tumors have been found in approximately 50% of patients.^5^^,^^6^^,^^8^ It has been reported that the male to female ratio of incidence is 2:1 and the age incidence is between 30 to 50 years old.^1^^,^^3^^,^^5^ Both our cases were male but age of one of them was out of this reported range. Most symptomatic patients have some degree of dysphagia, pain, pyrosis, weight loss, and weakness.^1^^-^^6^ Differential diagnoses include foreign body, malignant tumors of esophagus include squamous or adenomatous carcinomas or leiomyosarcoma and other benign tumors such as cysts and gastrointestinal stromal tumor (GIST).^5^

Leiomyoma is positive for desmin and actin and negative for CD34 and CD117 which oppositely occurs in GIST.^5^ Diagnostic work up begins with an upper GI endoscopy which shows a submucosal swelling with normal mucosa over it. Endoscopic ultrasonography (EUS), barium swallow, CT scan can help to differential diagnosis.^5^^,^^6^^,^^8^ Surgical enucleation is the accepted treatment for leiomyoma.^1^ The location of the lesions determines the side of the thoracoscopy: a left one is used for the lower third, whereas for lesions of the middle and upper thirds, a right thoracoscopy gives the best exposure and is widely used.^5^ The enucleation of leiomyoma through thoracoscopy was reported for the first time by Ohsawa in 1933.^2^^,^^5^

Until 1992, the enucleation of esophageal leiomyoma was traditionally performed via thoracoscopy.^2^^,^^5^ Everitt and colleagues and Bardini *et al.*, published the first reports of the thoracoscopic approach independently in 1992.^2^^,^^5^ The first published literature comparing open and minimally invasive surgery was presented by Von Rahden.^9^ The port sites and the number of used trocars are controversial issues. The thoracoscopic approach has been accomplished with seven ,^10^ six,^11^ five,^12^ four,^13^ and three trocars^2^^,^^14^^,^^15^ reported. However a 3-thoracic port technique which was deemed a safe procedure. Moreover, a thoracoscopy in the prone position has also been reported.^16^

Several different techniques have been described to assist extra mucosal enucleation using intraluminal tools. For example, esophageal SCT^17^ and a balloon dilator,^17^^,^^18^ have been employed and were found to be useful for facilitating the separation of the tumor by promoting progressive expulsion of the lesion from the esophageal wall. Izumi *et al.* expressed the use of a balloon-mounted esophagoscope as a new technique which called the balloon push-out method.^18^ This technique was suggested instead of pulling the tumor, which be hard to grasp due to its delicate nature and it was pushed out of the esophageal wall.^19^

We performed this surgery technique by using 3-ports without problems. Indeed protruding of mass by SCT assistance and hanging of mass by stitch help to reduced port, better dissection and faster operation. Although a successful procedure for large esophageal leiomyoma, which was removed by a thoracoscopic-endoscopic combined submucosal tunneling method was reported recently,^20^ but we never needed intraoperative endoscopy in this method. Indeed, for better exposure, dissection and detect the small mass we recommend use of esophageal SCT 40 F. For leakage test, we used injection of methylene blue by SCT instead NGT. It seems SCT is better than NGT due to large size of SCT. 

In case 1, we could not pass SCT at first due to large mass. Therefore, esophageal perforation occurred during dissection. If SCT was passed prior to dissection, perforation may not occur. The results of surgical treatment of esophageal leiomyoma have been generally excellent. Mortality rate after enucleation is low and success in relieving the dysphagia is about 100%. Few deaths, were reported following esophageal resection. The morbidity included some instances of either hemothorax or postoperative infection.^5^

Conclusively, the described technique is a safe, effective and feasible procedure with decreased hospital stay, operative trauma, reduced postoperative pain, quick recovery, and minute skin scars. Also, we used 3-port technique which is safe and effective. We describe here in a new videothoracoscopic technique to enucleate an esophageal leiomyoma with SCT assistance. The use of the SCT facilitates separation of the tumor mass interfering layers of esophagus, and may help to prevent perforation, and may help to detect of small mass which was not be detectable and operation was faster and more safely.
